# Dilute Species Transport During Generalized Newtonian Fluid Flow in Porous Medium Systems

**DOI:** 10.1029/2024wr037658

**Published:** 2025-01-04

**Authors:** Christopher A. Bowers, Cass T. Miller

**Affiliations:** 1 The University of North Carolina, Chapel Hill, NC, USA,; 2 North Carolina State University, Raleigh, NC, USA

## Abstract

Dilute species transport in generalized Newtonian fluids (GNFs) is typically described using explanatory empirical approaches assuming a traditional Fickian form, which is an approach that lacks predictive ability for systems and conditions not specifically investigated. Dilute species transport was investigated for a wide range of Cross and Carreau fluids flowing through a set of monodisperse and polydisperse sphere pack porous media. Both microscale and macroscale simulations were performed to demonstrate that GNF fluid flow can be predicted based upon Newtonian characterization of the media and rheological characterization of the fluid. Dilute species transport was shown to have a Fickian limit with dispersivity dependent on the porous media, fluid properties, and the flow rate in a nonlinear fashion. Dimensionless analysis and symbolic regression was used to deduce an explanatory and predictive function to describe dispersivity in terms of relevant system properties, enabling prediction of dilute species transport for GNFs flowing through porous media that does not require any non-Newtonian experiments or parameter estimation.

## 1. Introduction

The dispersion of a dilute solute through porous medium systems has been studied extensively due to its significance in biological, industrial, and environmental applications (Bear, 1972; Boccardo et al., 2020; Dentz et al., 2004; Domenico & Schwartz, 1998; Icardi et al., 2014; Sole-Mari et al., 2023; Taylor, 1953). Dilute species transport (DST) in non-Newtonian fluids is especially significant when modeling species transport in blood (Aruna & Barik, 2023; Dejam, 2018; Saadun et al., 2021; Singh & Murthy, 2022; Sochi, 2010, 2015) or toxic species in industrial applications such as hydraulic fracturing (An et al., 2022; Brown & Dejam, 2023; Buchley & Lord, 1973; Hauswirth et al., 2020). Hydraulic fracturing fluids in particular often contain many toxic additives at dilute levels, including 98 chemicals that were identified as toxic by the EPA (U.S. EPA, 2016), making an understanding of species dispersion in non-Newtonian fluids an important open question. Previous work has established that species dispersion in non-Newtonian fluids can depend upon the flow rate, but a general expression, even in the Fickian limit, has not been reported (Boschan et al., 2003, 2008; Chaplain et al., 1998; D’Onofrio et al., 1997, 2002; Dejam, 2018; Li et al., 2013; Paterson et al., 1996; Pearson & Tardy, 2002).

Non-Newtonian fluids exhibit a non-linear viscosity-shear stress relationship that can take the form of shear rate dependence, time dependence, viscoelasticity, or some combination of the three (Bird et al., 2002; Skelland, 1967). A commonly encountered class of non-Newtonian fluids is the generalized Newtonian fluid (GNF), which exhibits a viscosity that is dependent on shear rate, temperature, and pressure (C. A. Bowers & Miller, 2021; Hauswirth et al., 2020; Pearson & Tardy, 2002; Sorbie et al., 1989; Sochi, 2010; Zhang et al., 2019). The viscosity for a GNF is related to the shear stress acting on the fluid by a modified form of Newton’s law of viscosity given as

(1) $$\tau_{w}=2{\overset{ˆ}{\mu }}_{w}\left({\dot{\gamma }}_{w}\right)d_{w},$$

where $\tau_{w}$ is the viscous stress tensor, ${\overset{ˆ}{\mu }}_{w}$ is dynamic viscosity, which exhibits a functional dependence on the shear rate ${\dot{\gamma }}_{w}$ defined as

(2) $${\dot{\gamma }}_{w}=\sqrt{2d_{w}:d_{w}},$$

$d_{w}$ is the symmetric rate of strain tensor defined by

(3) $$d_{w}=\frac{1}{2}\left[\nabla v_{w}+{\left(\nabla v_{w}\right)}^{\text{T}}\right],$$

$v_{w}$ is velocity, subscripts denote that these are microscale, or pore-scale, quantities, w denotes the fluid phase, :d denotes the double dot product that contracts two tensors to a scalar defined in Einstein notation as $a:b=a_{\text{ij}}b_{\text{ji}}$, and T is the transpose operator (Bird et al., 2002; C. A. Bowers & Miller, 2021; Gray & Miller, 2014; Skelland, 1967; Sochi, 2010).

When modeling DST in porous media, there is a distinction between the pore-scale, which will be called the microscale, and the porous medium continuum scale at which the fluid and solid phases are not resolved separately, which will be referred to as the macroscale (Gray & Miller, 2014; C. A. Bowers & Miller, 2021). At the microscale, DST phenomena are separated into species advection and diffusion that may be considered independent from one another (Bear, 1972; de Marsily, 1986; Weigand et al., 2018). At the macroscale the predominant DST phenomena are advection and hydrodynamic dispersion, and these both depend on the averaged microscale velocity field (de Marsily, 1986; Icardi et al., 2014; Taylor, 1953). DST in GNFs where microscale velocities change non-linearly with respect to the average velocity provides an additional complication (D’Onofrio et al., 1997; C. A. Bowers & Miller, 2021; Singh & Murthy, 2022). There is currently no agreed upon macroscale model for DST during GNF flow, with past work carried out to quantify the difference between Newtonian and GNF dispersivity in real porous media without a final model being developed (D’Onofrio et al., 1997, 2002; Chaplain et al., 1998; Boschan et al., 2008; Li et al., 2013), or models being derived based on simplified fluid rheology during flow in idealized geometries such as capillary tubes (An et al., 2022; Boschan et al., 2008; Brown & Dejam, 2023; Chaplain et al., 1998). A desirable macroscale model would be one that can predict transport parameters based on known system and fluid characteristics for a range of porous media and is not based on system or fluid simplifications.

The goal of this work is to develop a macroscale model for DST in GNFs. The specific objectives are to: (a) to observe DST for GNFs flowing in microscale systems; (b) to upscale microscale results for DST to the macroscale; (c) to compare dispersion results for Newtonian and GNFs as a function of flow rates; (d) to develop a macroscale approximation for DST in GNFs in the Fickian limit; and (e) to evaluate the macroscale dispersion approximation for DST in GNFs as a function of flow rate.

## 2. Background

### 2.1. Generalized Newtonian Fluids

GNFs span a diversity of rheological behavior, with some common classes including power-law, Bingham, and Ellis fluids (Bird et al., 2002; Sochi, 2010). A class of fluid often encountered in industrial processes, including hydraulic fracturing, can be described using four parameter models that include Newtonian viscosities at high and low shear rates, as well as two parameters that define the transition between the two Newtonian viscosity limits; such fluids are often modeled using either a Cross or Carreau rheological model (Hauswirth et al., 2020; C. A. Bowers & Miller, 2021; Tosco et al., 2013; Zhang et al., 2019; Hauswirth et al., 2019; Basset et al., 2019; Perrin et al., 2006; Sochi, 2010; Sorbie et al., 1989; Tosco et al., 2013; Zami-Pierre et al., 2018; Rodríguez de Castro & Agnaou, 2019; Rodríguez de Castro & Goyeau, 2021).

Cross fluids exhibit rheology described by

(4) $${\overset{ˆ}{\mu }}_{w}\left({\dot{\gamma }}_{w}\right)={\overset{ˆ}{\mu }}_{\infty }+\frac{{\overset{ˆ}{\mu }}_{0}-{\overset{ˆ}{\mu }}_{\infty }}{1+{\left(m{\dot{\gamma }}_{w}\right)}^{n}},$$

where ${\overset{ˆ}{\mu }}_{\infty }$ is the viscosity limit as the shear rate approaches infinity, ${\overset{ˆ}{\mu }}_{0}$ is the viscosity limit as the shear rate approaches zero, m is a parameter that determines the shear rate at which non-Newtonian behavior becomes significant, and n is a non-Newtonian behavior index (Cross, 1965; Sochi, 2010; C. A. Bowers & Miller, 2021). Carreau fluids exhibit rheology described by (Bird et al., 2002; Sochi, 2010)

(5) $${\overset{ˆ}{\mu }}_{w}\left({\dot{\gamma }}_{w}\right)={\overset{ˆ}{\mu }}_{\infty }+\frac{{\overset{ˆ}{\mu }}_{0}-{\overset{ˆ}{\mu }}_{\infty }}{{\left[1+{\left(m{\dot{\gamma }}_{w}\right)}^{2}\right]}^{\frac{1-n}{2}}},$$

with rheological parameters that are similar to the Cross model described above but distinct for each model. Both the Cross and Carreau models exhibit an infinite and zero shear rate viscosity plateau at which they behave roughly as Newtonian fluids with a power-law like region that connects the two Newtonian limits, but the transition between these two limits has a different form for each model.

Four parameter rheological models, such as those discussed above, generate complex shear-rate profiles within porous medium systems and can complicate modeling efforts (Sorbie et al., 1989; Sochi, 2010; Tosco et al., 2013; Zami-Pierre et al., 2018; Hauswirth et al., 2020; C. A. Bowers & Miller, 2021; Rodríguez de Castro & Goyeau, 2021). In addition, calculation of the shear rate for Cross and Carreau model fluids can be numerically challenging (Bauer et al., 2019; Coussot, 2014; Spelt et al., 2005). Due to these reasons, Cross and Carreau fluids have often been modeled either as power-law fluids or as a power-law with a cut-off (An et al., 2022; Hauswirth et al., 2020; Zami-Pierre et al., 2016, 2018). However, such methods do not accurately model flow when fluids are transitioning between Newtonian plateaus (Boger, 1977; Chhabra & Uhlherr, 1979; Subbaraman et al., 1971), a transition which is often encountered during flow in porous medium systems. Thus, modeling efforts should be made to resolve the actual rheology observed for these fluids.

### 2.2. Flow Modeling

One defining feature of DST is that the species concentration does not impact the flow field of the bulk solvent, allowing bulk fluid flow to be modeled separately from species transport. For laminar flow of Newtonian fluids in porous medium systems, Darcy’s law is usually used to model the flow velocity as

(6) $$q^{\bar{\bar{w}}}=-\frac{{\hat{k}}^{w}}{{\hat{\mu }}^{w}}\cdot (\nabla p^{w}-p^{w}g^{\bar{w}}),$$

where $q^{\bar{\bar{w}}}$ is the specific discharge or Darcy velocity, defined by

(7) $$q^{\bar{\bar{w}}}=\epsilon^{\bar{\bar{w}}}v^{\bar{w}},$$

$\epsilon^{\bar{\bar{w}}}$ is volume fraction of the fluid phase, which is assumed equal to porosity $\epsilon ,{\overset{ˆ}{k}}^{w}$ is the permeability tensor of the medium, $p^{w}$ is the fluid pressure, $\rho^{w}$ is the mass density, $g^{\bar{w}}$ represents the gravitational acceleration vector, superscripts indicate averaged macroscale quantities, and w denotes the fluid phase (Gray & Miller, 2006, 2014).

It is apparent from Equations 4 and 5 that the viscosity observed during GNF flow can change dramatically from one flow rate to another, depending on the range of flow rates being investigated and the fluid properties as represented in the model parameters. It is also apparent that Darcy’s law, as it appears in Equation 6 with a constant viscosity, does not apply. In such cases, an effective viscosity, which is calculated using an empirically fitted shift factor, has historically been substituted into Equation 6 (C. A. Bowers & Miller, 2021; Hauswirth et al., 2020; Hayes et al., 1996; S. Liu & Masliyah, 1999; Perrin et al., 2006; Parnas & Cohen, 1987; Sadowshi & Bird, 1965; Sorbie et al., 1989; Tosco et al., 2013).

The effective viscosity approach to GNF flow modeling was developed phenomenologically and leads to a situation where empirical parameters must be fitted for each fluid of interest flowing through each system of interest, either using experimental or simulation data. Such data is not always available, hindering flow modeling efforts and thus making DST modeling impracticable.

Recent work has been carried out to model flow of both Cross and Carreau model fluids using a resistance model that only requires flow data from a Newtonian fluid flowing through a medium of interest and rheological properties of the GNF (C. A. Bowers & Miller, 2021; C. A. Bowers & Miller, 2023). The hydraulic resistance to flow is observed in the model below,

(8) $$-\epsilon^{\bar{\bar{w}}}\nabla p^{w}+\epsilon^{\bar{\bar{w}}}p^{w}g^{\bar{w}}+\epsilon^{\bar{\bar{ws}}}{\langle \rho_{w}\psi_{w}n_{w}\rangle }_{\Omega_{ws},\Omega_{\text{ws}}}={\hat{R}}^{w}\cdot v^{\bar{w}},$$

where $\epsilon^{\bar{\bar{\text{ws}}}}$ is specific interfacial area, $\psi_{w}$ is gravitational potential, $n_{w}$ is the outward normal of the w phase, ${\langle \;\rangle }_{\Omega_{\text{ws}},\Omega_{\text{ws}}}$ is the intrinsic averaging operator applied to the ws-interface, and ${\overset{ˆ}{R}}^{w}$ is the hydraulic resistance tensor (C. A. Bowers & Miller, 2021). During Newtonian fluid flow, the resistance may be calculated from

(9) $$\frac{{\hat{k}}^{w}}{{\hat{\mu }}^{w}}=\epsilon^{{\bar{\bar{w}}}^{2}}{\left({\hat{R}}^{w}\right)}^{-1},$$

while during Cross and Carreau model fluid flow, the resistance is computed from a resistance model that is based on the rheology of the fluid (C. A. Bowers & Miller, 2021; C. A. Bowers & Miller, 2023). Assuming a coordinate system is selected that leads to a diagonal resistance tensor, the diagonal entries of the resistance tensor may be computed during Cross model fluid flow from

(10) $${\hat{R}}_{\text{ii}}^{w}={\hat{R}}_{\infty \text{ii}}^{w}+\frac{{\hat{R}}_{0\text{ii}}^{w}-{\hat{R}}_{\infty \text{ii}}^{w}}{1+(M_{i}q_{i}^{\bar{\bar{w}}})},$$

where ${\overset{ˆ}{R}}_{\infty \text{ii}}^{w}$ is the iith-indexed entry of the Newtonian resistance tensor at ${\overset{ˆ}{\mu }}_{\infty },{\overset{ˆ}{R}}_{0\text{ii}}^{w}$ is the iith-indexed entry of the Newtonian resistance tensor at ${\overset{ˆ}{\mu }}_{0}$, and $M_{i}$ is a flow parameter that is computed from

(11) $$M_{i}=\frac{m{\hat{L}}_{\text{ii}}^{w}\sqrt{{\hat{R}}_{0\;\text{ii}}^{w}}{\hat{R}}_{\infty \text{ii}}^{w}}{\epsilon^{\bar{\bar{w}}}\sqrt{{\hat{\mu }}_{0}{\hat{\mu }}_{\infty }}},$$

where ${\overset{ˆ}{L}}_{\text{ii}}^{w}$ is the iith-indexed entry of the length scale tensor ${\overset{ˆ}{L}}^{w}$, which is a property of the medium (C. A. Bowers & Miller, 2021; C. A. Bowers & Miller, 2023). The length scale tensor is computed from

(12) $$-{\left\langle 2{\overset{ˆ}{\mu }}_{w}d_{w}\cdot n_{w}\right\rangle }_{\Omega_{\text{ws}},\Omega_{\text{ws}}}={\overset{ˆ}{L}}^{w}\cdot {\overset{ˆ}{R}}^{w}\cdot v^{\bar{w}},$$

and only requires data from one single Newtonian fluid flowing through the system of interest (C. A. Bowers & Miller, 2021). A relationship between the length scale tensor and system properties has recently been derived, and it was found that the tensor is related to porosity, Newtonian permeability, mean curvature, and Gaussian curvature for several classes of porous medium systems (C. A. Bowers & Miller, 2023). Similarly to Cross model fluid flow, the entries of the resistance tensor during Carreau model fluid flow may be calculated from

(13) $${\hat{R}}_{\text{ii}}^{w}={\hat{R}}_{\infty \text{ii}}^{w}+\frac{{\hat{R}}_{0\text{ii}}^{w}-{\hat{R}}_{\infty \text{ii}}^{w}}{{\left[1+{\left(M_{i}q_{i}^{\bar{\bar{w}}}\right)}^{2}\right]}^{\frac{1-n}{2}}},$$

where all parameters are computed identically to the Cross-like resistance model (C. A. Bowers & Miller, 2023).

The resistance models discussed above were developed based on insights gained from analysis using thermodynamically constrained averaging theory (TCAT) (C. A. Bowers & Miller, 2021). These models, as well as the length-scale relationship developed in (C. A. Bowers & Miller, 2023), may be used to model flow of Cross and Carreau model fluids in porous media using known or easily tabulated system parameters, as well as rheological parameters for each fluid of interest. This puts flow modeling of GNFs on an even footing with methods used to model flow of Newtonian fluids and provides a convenient way to model flow of GNFs as a component of analyzing DST.

### 2.3. Dilute Species Transport Modeling

DST has been extensively studied for Newtonian fluid flow in a variety of systems (Bear, 1972; Bird et al., 2002; Boccardo et al., 2020; de Marsily, 1986; Dentz et al., 2004; Domenico & Schwartz, 1998; Icardi et al., 2014; Sole-Mari et al., 2023; Taylor, 1953). The classical approach to modeling DST for a constant porosity system at the macroscale utilizes the advection-dispersion equation, given for an inert species i by

(14) $$\frac{\partial \left(\rho^{w}\omega^{i\;\bar{w}}\right)}{\partial t}+v^{\bar{w}}\cdot \nabla \left(\rho^{w}\omega^{i\;\bar{w}}\right)-\nabla \cdot \left[{\overset{ˆ}{D}}^{w}\cdot \nabla \left(\rho^{w}\omega^{i\;\bar{w}}\right)\right]=0,$$

where $\omega^{i\;\bar{w}}$ is the mass fraction for species i and ${\overset{ˆ}{D}}^{w}$ is the hydrodynamic dispersion tensor (de Marsily, 1986). For unidirectional flow aligned with the coordinate system, Fickian dispersion can be described as a diagonal tensor dependent upon a longitudinal dispersion coefficient in the direction of flow, ${\overset{ˆ}{D}}_{L}^{w}$, and transverse dispersion coefficients ${\overset{ˆ}{D}}_{T}^{w}$ for directions orthogonal to the flow direction (Bear, 1972; de Marsily, 1986). In the case that the coordinate system is not aligned with the direction of flow, an Eigenvalue decomposition can be carried out on ${\overset{ˆ}{D}}^{w}$, with eigenvalues corresponding to one longitudinal and two transverse dispersion coefficients. During Newtonian fluid flow, the longitudinal dispersion has historically been computed from a linear relationship to velocity, given as

(15) $${\overset{ˆ}{D}}_{L}^{w}={\overset{ˆ}{\alpha }}_{L}^{w}\left|v^{\bar{w}}\right|+\frac{{\overset{ˆ}{D}}_{m}}{{\overset{ˆ}{\tau }}^{w}},$$

where ${\overset{ˆ}{\alpha }}_{L}^{w}$ is the longitudinal dispersivity, which may be considered a property of the medium for the Newtonian case, ${\overset{ˆ}{D}}_{m}$ is the molecular diffusion, and ${\overset{ˆ}{\tau }}^{w}$ is the tortuosity of the medium (Bear, 1972; de Marsily, 1986; Domenico & Schwartz, 1998; Weigand et al., 2018). In the case that $v^{\bar{w}},{\overset{ˆ}{\alpha }}_{L}^{w}$, and ${\overset{ˆ}{\tau }}^{w}$ are known, a one-dimensional form of Equation 14 becomes a closed model for DST during Newtonian fluid flow in the longitudinal direction for a homogeneous, isotropic media at length scales above those needed for Fickian behavior.

Classical dispersion theory as described above has led to an extensive literature where dispersivity coefficients are computed by observing transport through a single porous medium in the laboratory, and these dispersivities are then tabulated for the medium (Delgado, 2006; Freeze & Cherry, 1979; Kitanidis, 1988; Y. Liu & Kitanidis, 2013; Maineult et al., 2016; Sharma & Abgaze, 2015). However, during DST in geological formations, it has been recognized that there is a local scale for which the porous medium is roughly homogeneous, at which scale linear Fickian dispersion phenomena are observed, and a non-local scale at which the medium is heterogeneous and classical dispersion theory does not apply (Dagan, 1984; Dentz et al., 2002; Di Federico & Neuman, 1998; Morales-Casique et al., 2006; Neuman & Zhang, 1990). Linear Fickian dispersion may be recovered as the distance of observation is increased to sufficient scales, exhibiting a phenomenon which has been called macrodispersion, but these scales are often larger than the scale of a useful modeling domain (Dagan, 1984; Dagan & Lessoff, 2001; Hansen et al., 2018; Meyer, 2018; Stettler et al., 2023). Non-local models are necessary when modeling DST during flow in formations that exhibit heterogeneity in permeability. While future modeling efforts will need to be made to incorporate GNF rheology into non-local dispersivity theory, accurate models at even the local scale are not known for significant classes of GNFs, such as those investigated here. As a first step toward the goal of DST modeling during GNF flow in real geological formations, this work will focus on local scales where the linear Fickian limit may be reasonably expected to be reached and where classical dispersion theory applies.

Local-scale modeling of DST during GNF flow in porous media at the macroscale is significantly less mature than for the Newtonian case. During GNF flow, the microscale velocity field is not linearly related to the average velocity in the way that it is for Newtonian fluid flow (C. A. Bowers & Miller, 2021), and thus the hydrodynamic dispersion is not expected to exhibit linear behavior either (Chaplain et al., 1998; D’Onofrio et al., 2002; Saadun et al., 2021; Singh & Murthy, 2022). Past efforts to model DST during GNF flow have focused on quantifying the change in dispersivity from the Newtonian case, after which point transport could be modeled using Equation 14, although this is not predictive outside the observed range (An et al., 2022; Brown & Dejam, 2023; Boschan et al., 2003, 2008; Dejam, 2018; D’Onofrio et al., 1997; Li et al., 2013). Furthermore, if a predictive transport model were developed heretofore, it would require a predictive GNF flow model that did not require additional simulation or experimental data, which has not appeared in the literature prior to (C. A. Bowers & Miller, 2021; C. A. Bowers & Miller, 2023) A desirable model would be able to predict the dispersivity at a given flow rate using known system and rheological parameters such that a closed transport model is achieved without the need to carry out experiments for each fluid and velocity of interest.

## 3. Methods

### 3.1. Overview

Methods are desired to confirm that DST in GNF deviates from Newtonian systems and, if so, to determine quantitatively if it tends to a Fickian limit. It is further desirable to formulate a quantitative approximation that describes the observed behavior that can be used to predict DST in GNF systems not specifically investigated. To meet these objectives, a microscale computational approach that is averaged to the macroscale was used. The systems considered, microscale modeling methods, macroscale modeling methods, and statistical analysis methods used are presented in turn in the sections that follow.

### 3.2. Porous Medium Systems

**Figure 1.**
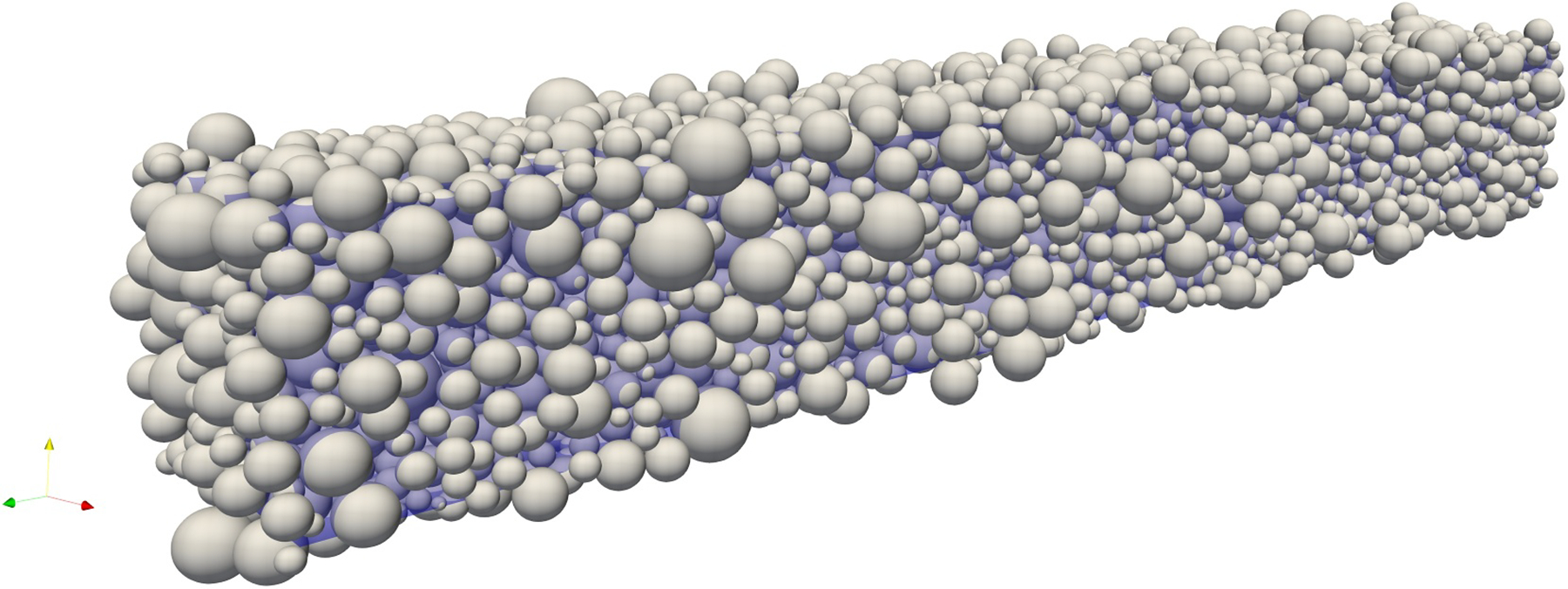
Random sphere packing with a log-normal particle radius variance of 0.1.

Given the state of development of modeling DST in GNFs, sphere pack systems were used for the investigation, which is a common choice for model systems (Boccardo et al., 2020; C. A. Bowers & Miller, 2021; Hauswirth et al., 2020; Sole-Mari et al., 2023; Weigand et al., 2018). The sphere packs were generated using a log-normal distribution of spheres arranged in a non-overlapping, gravitationally stable arrangement using an existing algorithm (Williams & Philipse, 2003). Four sphere packings were used with log-normal variances that increased from 0 to 0.1 in order to generate data for a range of pore morphologies. The system properties are described in Table 1. The largest log-normal variance was selected based on what may be considered a relatively non-uniform medium, while the average radii of the spheres were consistent with a fine-grained sand (Hauswirth et al., 2020). The geometry of the domain was based upon the desire to test the Fickian assumption and domains used previously for similar purposes (Weigand et al., 2018). The large variance sphere packing used is depicted in Figure 1. The other media used are more uniform than the example shown.

Four Cross fluids and four Carreau fluids were simulated so that each of the four parameters in each model could be varied. The parameters used for the four test fluids of each model are defined by Table 2. When referring to fluids from Table 2, Cross Fluid 1 and Carreau Fluid 1 both have the same parameters, with the model being switched between Equations 4 and 5. The order of magnitude of the zero and infinite shear limit viscosities of Fluids 1 were selected to be similar to what is seen in guar gum, as shown in Hauswirth et al. (2020), while the n parameters chosen are close to what is typically used (Hauswirth et al., 2020; Zhang et al., 2019). The m parameters used for all fluids were selected to ensure that the full range of GNF behavior can be observed in the systems being investigated during laminar flow. Following conventions common in the GNF literature, each of the four rheological model parameters are independently varied to isolate the impact each parameter has on the model (C. A. Bowers & Miller, 2021; C. A. Bowers & Miller, 2023; Lenci et al., 2022; Sochi, 2015; Zhang et al., 2019). In the next sections, the specific numerical methods used are described.

### 3.3. Simulation Overview

Flow and transport simulations were carried out for all porous media investigated here, with a difference in the flow rate and fluid resolution between different media. For the highest variance medium, all of the fluids were simulated and a fine degree of flow rate resolution was achieved. This was done to determine if Fickian behavior existed for a bounding case for this class of media and to obtain a high-resolution basis to determine how dispersivity changes with flow rate and rheological properties. Other media were investigated to evaluate how medium properties impact dispersivity during GNF flow, and fewer flow rates were simulated to reduce the computational burden. Additionally, only Carreau Fluid 3, and Cross Fluids 1 and 3 were simulated for the more uniform media. Rheological parameters for Fluid 3 were selected as bounding rheological cases, while Cross Fluid 1 was selected as representative of the other fluids.

### 3.4. Microscale Simulation

The systems simulated included steady-state isothermal, Stokes flow of either a Cross or a Carreau GNF through the porous media described above. The geometry of all solid particles was resolved at the microscale and the solution involved a finely resolved fluid velocity and the transport of a dilute species within the GNF that was assumed to not affect the fluid density or viscosity. Species were assumed to not undergo mass transfer between phases; the density of the fluid was assumed to be constant; and the solid phase was assumed to be non-deformable and non-mobile—yielding a constant volume fraction in space at the macroscale.

Microscale simulations involved mesh generation, steady-state flow field simulations to determine the microscale velocity field, and species transport simulations. The flow and transport simulations were uncoupled because the density and viscosity of the fluid did not depend upon the transport of the dilute species. Simulations were performed using available OpenFOAM packages (Greenshields, 2018).

A background mesh was generated using the blockMesh utility and was then manipulated to include the ws-interface using the snappyHexMesh utility (Greenshields, 2018). A level-one near-solid refinement was used with snappyHexMesh, which has been found to yield efficient, resolved solutions for similar systems in OpenFOAM (Icardi et al., 2014; Weigand & Miller, 2020; C. A. Bowers & Miller, 2021, 2023). The refinement of the background mesh was determined first by carrying out flow simulations through the porous medium of Carreau Fluid 1 at multiple flow rates, using subsequently increased mesh refinements, and comparing the resulting hydraulic resistance to the result of the previous refinement. Each subsequent mesh refinement included double the number of background cells in each dimension compared to the previous refinement. Once the hydraulic resistance was found to change by less than 1% relative to the previous refinement, the simulation was considered sufficiently resolved to be grid-independent for flow simulation. The hydraulic resistance was predicted using Equation 13 for the grid-resolved mesh and the flow rate at which the inflection-point in the log of the resistance occurred was determined. The greatest error observed during flow simulation has occurred at this inflection-point flow rate (C. A. Bowers & Miller, 2021; C. A. Bowers & Miller, 2023), and thus this flow rate was used to test convergence behavior during transport simulation as an upper error bound.

To determine grid-independence for transport simulations, meshes were again generated using subsequently increasing refinement and flow and transport simulations were carried out. The dispersivity observed during these simulations was computed, as described in the next section. Once a mesh achieved a dispersivity that changed by less than 1% relative to the last mesh refinement, it was determined that the mesh had achieved grid independence for transport simulation. The higher mesh refinement required to achieve grid-independence between flow and transport simulations was selected. The mesh refinement level that achieved grid-independence for flow simulation was similar to what was observed in (C. A. Bowers & Miller, 2021; C. A. Bowers & Miller, 2023) when comparing number of cells-per-mean sphere diameter. The discretization scheme used resulted in the values in Table 1, which were used for both flow and transport simulations.

Microscale flow simulations were carried out using the simpleFOAM modeling package (Greenshields, 2018). This package uses the SIMPLE algorithm (Caretto et al., 1972) to solve the microscale incompressible conservation of mass and momentum equations for a phase, defined as

(16) $$\nabla \cdot v_{w}=0,$$

and

(17) $$\nabla \cdot \left(v_{w}v_{w}\right)-\nabla \cdot_{w}=-\nabla p_{w}+S,$$

where S is a momentum source term. For Cross and Carreau fluids, ${\overset{ˆ}{\mu }}_{w}$ depends upon the shear rate, which was computed using Equations 2 and 3 (Greenshields, 2018).

The flow through the porous medium was driven by a body-force S that was aligned with the z dimension to drive flow in that direction. Zero-gradient boundary conditions were imposed for $p_{w}$ at the ws-interface and at the domain boundaries, a zero-gradient boundary condition was imposed in $v_{w}$ at the domain boundaries, and a noslip boundary condition was enforced at the ws-interface. A pressure of zero was set for one cell by OpenFOAM, which was used as a reference for other pressures within the system to ensure a unique solution.

Microscale DST simulations were performed using the scalarTransportFOAM package built into OpenFOAM during transport of an arbitrary species A. The scalarTransportFOAM utility solves an advection-diffusion equation, which may be written as

(18) $$\rho_{w}\frac{\partial \omega_{\text{Aw}}}{\partial t}+\rho_{w}\nabla \cdot \left(v_{w}\omega_{\text{Aw}}\right)-\rho_{w}\nabla \cdot \left({\overset{ˆ}{D}}_{m}\nabla \omega_{\text{Aw}}\right)=S_{C},$$

where ${\overset{ˆ}{D}}_{m}$ is the molecular diffusion coefficient, and $S_{C}$ is a source term (Greenshields, 2018). The initial condition for the system was such that the dilute species being modeled was absent from the system so that

(19) $$\omega_{\text{Aw}}(t=0,x,y,z)=0.$$

A Dirichlet mass fraction boundary condition at the inlet (z=0) was set at 1 × 10^−6^, while a zero-gradient boundary condition was set for the outlet ($z=L_{z}$), lateral boundaries, and the ws-interface. Equation 18 was solved using a backward Euler method for the time derivative, a second-order centered finite difference method for the diffusive term, and a linear upwind method for the advective term. $D_{m}$ was chosen such that the Pe was always greater than 1,000, defined here as

(20) $$\text{Pe}=\frac{v^{\bar{w}}L_{z}}{{\overset{ˆ}{D}}_{m}}>1,000,$$

where $L_{z}$ is the length in the z-dimension (An et al., 2022; Boschan et al., 2008; Chaplain et al., 1998). Finally, the velocity field was taken from the simpleFOAM flow simulation. The area weighted average mass fraction at the outlet was calculated using the patchAverage utility built into OpenFOAM (Greenshields, 2018).

The microscale flow simulations were carried out on 216 computing cores on the Dogwood computing cluster at the University of North Carolina at Chapel Hill. The microscale DST simulations were carried out on 1,728 computing cores on the Dogwood computing cluster at the University of North Carolina at Chapel Hill. The averaged quantities calculated from these microscale simulations were used in conjunction with Equations 8, 12 and 14 to carry out macroscale analyses.

### 3.5. Macroscale Analysis

One of the primary questions that this work sets out to answer is whether a linear Fickian relationship can be applied to DST during GNF flow. To answer this question, the averaged data from microscale simulations were compared to macroscale simulations that make use of the linear Fickian assumption. For this case, flow was in one-dimension through an isotropic medium, which can be described with a classical Fickian transport equation for an arbitrary species A of the form

(21) $$\frac{\partial \omega^{A\;\bar{w}}}{\partial t}+v_{z}^{w}\frac{\partial \omega^{A\;\bar{w}}}{\partial z}-{\overset{ˆ}{D}}_{L}^{w}\frac{\partial^{2}\omega^{A\;\bar{w}}}{\partial z^{2}}=0,$$

where $v_{z}^{w}$ is the average velocity in the z-dimension, and ${\overset{ˆ}{D}}_{L}^{w}$ is a longitudinal hydrodynamic dispersion coefficient. This equation was solved numerically using a cell-centered finite difference scheme for the gradient and Laplacian, and a backwards Euler method for the time derivative, which was sufficiently accurate for the highly resolved discretizations used in this work (Weigand et al., 2018; Weigand & Miller, 2020). Comparing to the mass fraction computed by averaging microscale data at the outlet of the system, the dispersion coefficient was fitted to Equation 21 using the lsqcurvefit non-linear least squares solver in Matlab (MathWorks, 2022). The root-squared-mean-error (RSME) was then determined by comparing $\omega^{A\;\bar{w}}$ computed from Equation 21 to averaged microscale mass fraction data. The RMSE computed in this way gives an indication of whether the flow is Fickian because Equation 21 makes use of the linear Fickian assumption. This RMSE was also compared to the RMSE observed during Newtonian flow through the system, which is known to be Fickian, to determine how far observed behavior was from the Fickian limiting case.

The longitudinal dispersivity, ${\overset{ˆ}{\alpha }}_{L}^{w}$, was computed by re-arranging Equation 15, giving

(22) $${\overset{ˆ}{\alpha }}_{L}^{w}=\frac{1}{v_{z}^{w}}\left({\overset{ˆ}{D}}_{L}^{w}-\frac{{\overset{ˆ}{D}}_{m}}{{\overset{ˆ}{\tau }}^{w}}\right).$$

This is in keeping with what is typical in the literature, where the dispersivity observed during GNF flow is compared to the Newtonian dispersivity to quantify the impact of GNF behavior (An et al., 2022; Brown & Dejam, 2023; Boschan et al., 2008; Chaplain et al., 1998; D’Onofrio et al., 1997, 2002; Li et al., 2013). The dispersivity data observed for each of the GNFs investigated here was used to derive an approximation of ${\overset{ˆ}{\alpha }}_{L}^{w}$. A useful approximation would provide a sufficiently accurate prediction of the dispersivity without the solution of an inverse problem for the specific fluid properties and flow conditions of interest. Statistical analysis was used to meet this objective, which is discussed in the section that follows.

### 3.6. Statistical Analysis

To analyze the macroscale dispersivity data, a dimensional analysis was carried out, followed by statistical analysis to develop a functional form for the dispersivity model. Before beginning a dimensional analysis, it must be noted that there are two different classes of GNF being investigated here and that any statistical analysis started in this state would necessarily need to be siloed into a Cross fluid and a Carreau fluid analysis. To utilize data from both classes of fluids, a combined hydraulic resistance model based on Equations 10 and 13 was used. This combined model, which is a scalar here due to isotropic system conditions, is

(23) $${\overset{ˆ}{R}}^{w}={\overset{ˆ}{R}}_{\infty }^{w}+\frac{{\overset{ˆ}{R}}_{0}^{w}-{\overset{ˆ}{R}}_{\infty }^{w}}{{\left[1+{\left(Mq^{\bar{\bar{w}}}\right)}^{n_{1}}\right]}^{\frac{1-n_{2}}{2}}},$$

where $n_{1}$ is n for Cross fluids and 2 for Carreau fluids, and $n_{2}$ is −1 for Cross fluids and n for Carreau fluids. In addition, it is useful to expressly compute the change in resistance with respect to the Darcy velocity for this combined model, as this may be a useful predictor of the non-Newtonian character at a given flow rate (C. A. Bowers & Miller, 2023). Taking the derivative of Equation 23 with-respect-to $q^{\bar{\bar{w}}}$ gives

(24) $$\frac{\text{d}{\overset{ˆ}{R}}^{w}}{\text{d}q^{\bar{\bar{w}}}}=-\frac{n_{1}\left(1-n_{2}\right)\left({\overset{ˆ}{R}}_{0}^{w}-{\overset{ˆ}{R}}_{\infty }^{w}\right){\left(Mq^{\bar{\bar{w}}}\right)}^{n_{1}}}{2q^{\bar{\bar{w}}}{\left[1+{\left(Mq^{\bar{\bar{w}}}\right)}^{n_{1}}\right]}^{\frac{3-n_{2}}{2}}},$$

which will be used in the following analysis.

A candidate variable set was selected for dimensional analysis, given below

(25) $$𝒱=\left\{q^{\bar{\bar{w}}},{\overset{ˆ}{R}}^{w},{\overset{ˆ}{R}}_{\infty }^{w},{\overset{ˆ}{R}}_{0}^{w},M,n_{1},n_{2},\frac{\text{d}{\overset{ˆ}{R}}^{w}}{\text{d}q^{\bar{\bar{w}}}},{\overset{ˆ}{\alpha }}_{L}^{w},{\overset{ˆ}{\alpha }}_{L0}^{w}\right\},$$

where ${\overset{ˆ}{\alpha }}_{L0}^{w}$ is the longitudinal dispersivity when a Newtonian fluid is flowing through the system. Note that the variable set did not include parameters that may be expected to impact dispersivity, such as $\epsilon^{\bar{\bar{w}}},\epsilon^{\bar{\bar{\text{ws}}}}$, and ${\overset{ˆ}{L}}^{w}$. This is because either the variables did not change significantly from one medium to the next, or the impacts were expected to be expressed in the other quantities selected. Based on Equation 25, a Buckingham Pi analysis was performed yielding the non-dimensional equation

(26) $$\frac{{\overset{ˆ}{\alpha }}_{L}^{w}}{{\overset{ˆ}{\alpha }}_{L0}^{w}}=F\left(Mq^{\bar{\bar{w}}},\frac{{\overset{ˆ}{R}}^{w}}{{\overset{ˆ}{R}}_{\infty }^{w}},\frac{{\overset{ˆ}{R}}^{w}}{{\overset{ˆ}{R}}_{0}^{w}},n_{1},n_{2},\frac{q^{\bar{\bar{w}}}}{{\overset{ˆ}{R}}^{w}}\frac{\text{d}{\overset{ˆ}{R}}^{w}}{\text{d}q^{\bar{\bar{w}}}}\right),$$

where F is an unknown functional form representing a function that maps the non-dimensional groups on the right-hand-side of the equation to the non-dimensional variable group on the left-hand-side of the equation. Given the selection of the candidate set of variables, Equation 26 is the simplest possible functional form. However, some variables may be of lesser importance than the most important leading-order dimensionless quantities. Identifying F using traditional trial-and-error approaches for a system with seven independent non-dimensional quantities is a daunting task.

While Buckingham Pi analysis reduces the number of variables to the minimum number required to develop a function, the form of the function is not known from this analysis. To query the space of possible functions that may be used in Equation 26, statistical analysis was carried out based on symbolic regression (SR) (Cranmer, 2023). SR is the act of searching for a statistical model in a space of analytical expressions that are physically meaningful to a problem. PySR, a machine-learning SR approach, was used to identify candidate forms of F in this work (Cranmer, 2023). PySR is an open-source python library that combines machine-learning with SR algorithms.

The PySR algorithm operates by randomly varying branches of a symbolic expression tree and scoring the function generated from this tree based on function complexity and the loss, or error, generated by the resulting expression (Cranmer, 2023). The machine-learning algorithm then selects which expression trees are used in the next generation of mutation based on the scores of each tree, creating ever improving expressions until a stopping condition is met. Complexity is defined by default to be the sum of the number of constants, variables, and operations in a function; however, these may be altered based on the interpretability of the resulting expressions for each use case (Cranmer, 2023). Table 3 lists the controls used during PySR analysis. Most of these controls are related to algorithm performance, but a few deserve discussion. The operators selected were based on typical operations carried out in fluid mechanics. The complexity of constants was increased to encourage the algorithm to search for functions that utilized all the input variables. The exponentiation operator was more severely constrained so that it would be used sparingly and so that exponent quantities were not overly complex. Exponentiation has been singled out as an operator that requires constraint to generate interpretable functions using the PySR algorithm (Cranmer, 2023). The max complexity was selected based on the numerical error in the microscale simulations. The loss function used was the relative error percentage between the predicted and observed dispersivities.

## 4. Results

### 4.1. Overview

Flow and species transport simulations were carried out for each porous medium. The hydraulic resistances observed during flow simulations are discussed first, as well as the observed error between simulation and analytical resistance model results. After this, the species transport simulation results are discussed, and dispersivities fitted for the macroscale transport model are presented. The dispersivity data observed for all four porous media are then analyzed using the PySR symbolic regression algorithm, and a set of dispersivity models are presented.

### 4.2. Flow Simulation

Flow simulations were carried out over a range of flow rates such that the full range of GNF behavior was observed. The resistance was computed from Equation 8 after computing the average velocity within the medium (C. A. Bowers & Miller, 2021). Additionally, ${\overset{ˆ}{L}}^{w}$ was computed from Equation 12, and resistance parameters were computed from Equations 9 and 11 for each fluid in each medium using the system properties given in Table 4. The error between the observed hydraulic resistance and the hydraulic resistance computed from Equations 10 and 13 was highest for Packing 4, and thus the data from this packing will be highlighted and discussed specifically. This is also the medium for which flow rate resolution is the highest.

**Figure 2.**
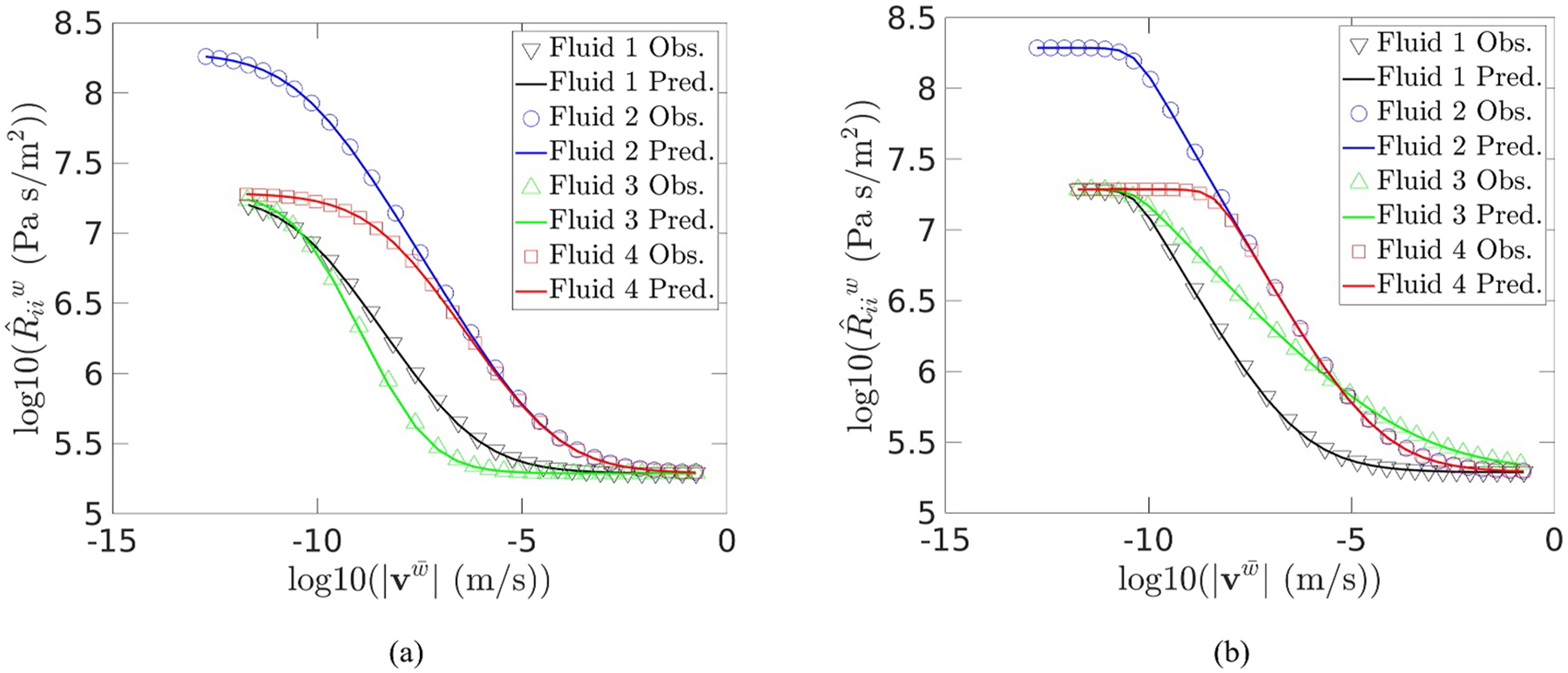
Hydraulic resistance observed and predicted during flow in the Packing 4 for: (a) Cross fluids; and (b) Carreau fluids.

The hydraulic resistance data for Packing four is shown in Figure 2, where it agrees with the resistances computed from Equations 10 and 13. The average relative errors between the predicted and observed hydraulic resistances shown in Figure 2 were between 1.1% and 1.9%, with most fluids exhibiting an average relative error of 1.5%. The maximum error observed was 3.8%, which occurred near the inflection point of the log of hydraulic resistance relative to velocity, as has been previously reported (C. A. Bowers & Miller, 2021; C. A. Bowers & Miller, 2023). The class of fluid did not significantly impact the average errors. The errors observed in the predicted resistance values are of similar order to the numerical errors of the simulations themselves, which were on the order of 1%, as discussed in § 3. These results provide additional validation of the previously derived approaches to predict GNF flow based upon only the Newtonian resistance and the rheological parameters of the fluid.

### 4.3. Macroscale Species Transport

**Figure 3.**
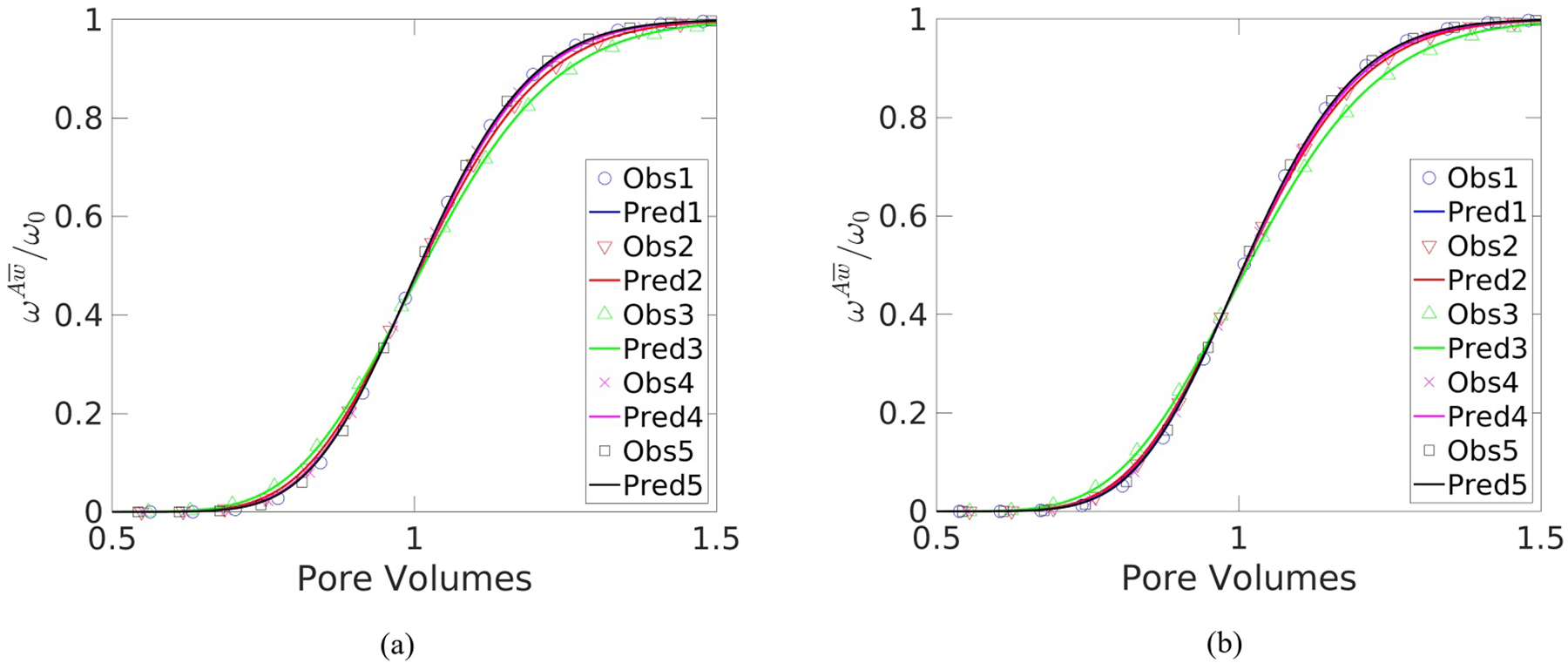
Select breakthrough curves comparing simulated to predicted results for a set of different specific discharges in Packing 4 for: (a) Cross Fluid 2; and (b) Carreau Fluid 2.

Velocity fields were used to carry out microscale species transport simulations for each medium. The average mass fraction at the effluent of the system was then used to fit a dispersion coefficient to Equation 21. To compare the Fickian-based macroscale model to the averaged microscale simulation results, breakthrough curves that display the effluent mass-fraction normalized by $\omega_{0}$ versus the number of pore-volumes passed through the system were generated. The breakthrough curves for five flow rates that represent the full spectrum of observed behavior are displayed in Figure 3 for Cross and Carreau Fluids 2, flowing through Packing 4. The data for these fluids flowing in this medium was selected because they exhibited the highest error between the macroscale model and the observed results.

It may be seen from Figure 3 that the microscale simulation results and macroscale model compare favorably. The breakthrough curves were equivalent to the Newtonian curve at low flow rates and became more disperse as the flow rate increased until an inflection occurred, at which point the curves became less disperse until they reached the Newtonian limit again. The RMSE between the Fickian macroscale model and microscale simulation results for Newtonian fluid flow was roughly 1.8% for all media, which was the benchmark used to determine whether Fickian behavior applies to the GNFs at varying flow rates. The maximum RMSE observed increased as the variance in sphere radii increased, with an RMSE range of 1.8%–2.5% for the monodisperse medium, and a range of 1.8%–3.0% for Packing 4. The maximum RMSEs occurred for each media when the inflection point in the log of resistance occurred. The average RMSE was 2%, which is on the order of the expected error of the transport simulations, which was roughly 1%, indicating that the Fickian assumption is a reasonable one for GNF flow for the media investigated here.

**Figure 4.**
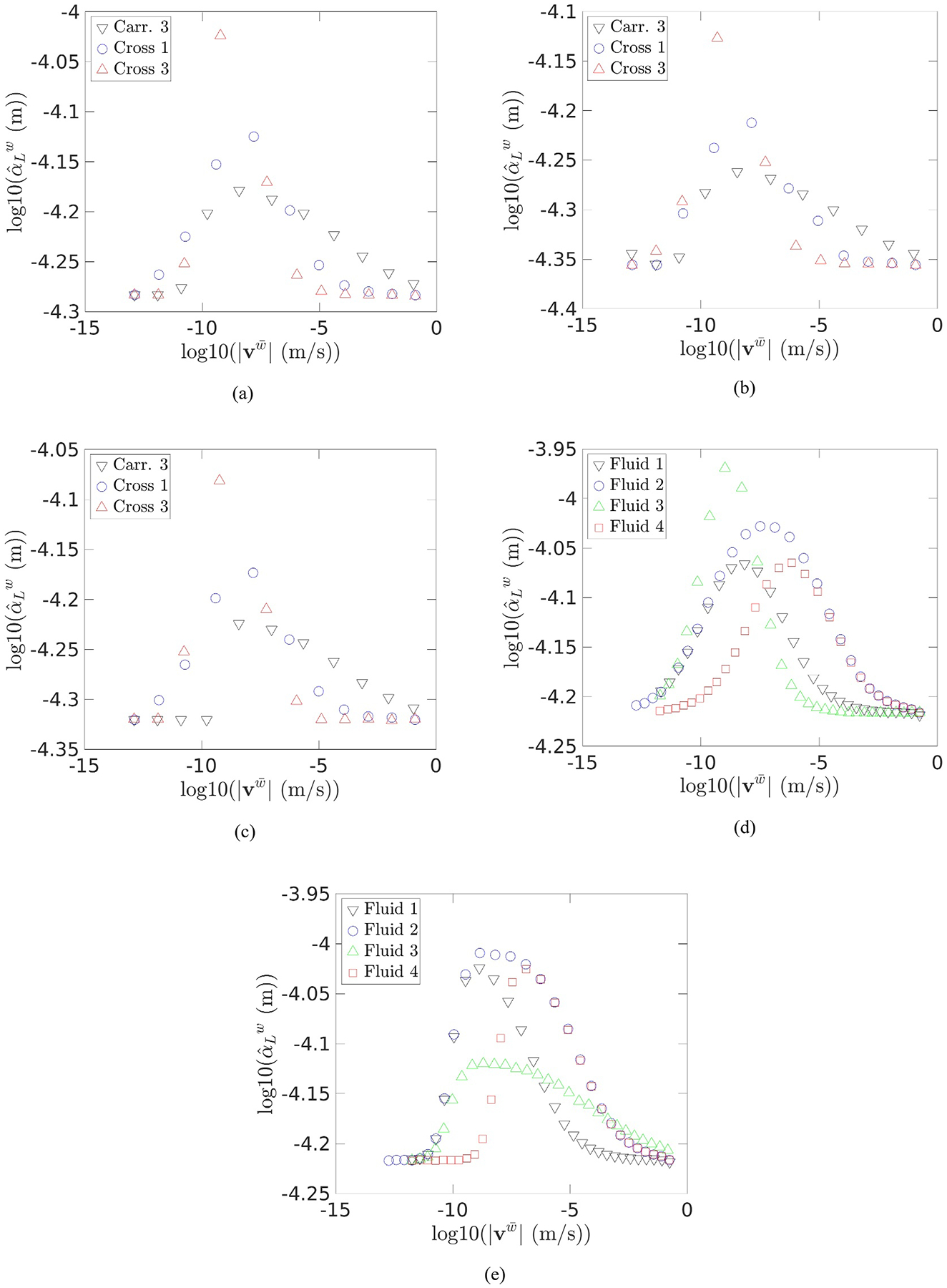
Dispersivity observed during DST for: (a) Packing 1; (b) Packing 2; (c) Packing 3; (d) Cross fluids in Packing 4; and (e) Carreau fluids in Packing 4.

To quantify how much dispersion changes with specific discharge for different GNFs, the longitudinal dispersivity was computed from Equation 22 for each fluid at each flow rate. The dispersivities for the Cross and Carreau fluids are plotted as a function of velocity in log-space in Figure 4. Similarly to the qualitative assessment of the breakthrough curves given above, the dispersivity was observed to increase from the Newtonian dispersivity up to a maximum, then decrease back to the original Newtonian dispersivity. The dispersivity changed by up to 1.8 times the Newtonian dispersivity, which is consistent with what has been observed in the literature (D’Onofrio et al., 2002; An et al., 2022). The dispersivities appear to exhibit curves that correspond to their rheological fluid parameters, motivating investigation to determine a function to describe dispersivity on this basis similar to what was done for hydraulic resistance in C. A. Bowers and Miller (2021). If a function for dispersivity with respect to velocity can be derived, then a closed macroscale model for DST during GNF flow will have been achieved.

### 4.4. Statistical Analysis

The dispersivity data generated by fitting the macroscale transport model to averaged microscale simulation data was analyzed using the PySR algorithm as described in § 3. Data generated during simulation in all four porous media were used during analysis, resulting in 323 data points. When selecting a dispersivity model, the numerical error present during microscale simulations, as well as the error between the microscale data and macroscale transport model were considered.

The summary of optimal functions output by PySR for each level of allowed complexity is listed in Table 5, along with the corresponding loss and generalized cross validation (GCV) value computed. Recall that the loss function is the average relative error percentage, as described in § 3.6. The GCV is a rotational invariant form of the ordinary cross-validation, and is measured using the mean-squared error, as described in (Golub et al., 1979). It may be noted that, although all of the dimensionless quantities that appear in Equation 26 were included in the PySR analysis, not all of these variables appear amongst the equations that exhibited the lowest loss. An inspection of Table 5 reveals that $\left(q^{\bar{\bar{w}}}/{\overset{ˆ}{R}}^{w}\right)\text{d}{\overset{ˆ}{R}}^{w}/\text{d}q^{\bar{\bar{w}}}$ appears in every function that includes variables. The only other quantity that appears with regularity is ${\overset{ˆ}{R}}^{w}/{\overset{ˆ}{R}}_{0}^{w}$, indicating that the resistance is the most significant factor when calculating dispersivity, and especially the change in resistance with-respect-to flow rate. It should also be noted that all functions listed in Table 5 that include variables achieve Newtonian limits at very high and very low velocities as expected based on Figure 4.

When selecting a functional form to utilize going forward, the complexity, loss, and human interpretability of the function need to be evaluated. Functions 2–7 all achieved an average loss of less than 2%, making any of these candidates to model dispersivity during GNF flow. Function four is an intuitive functional form that includes only one exponent quantity, which may be written as

(27) $$\frac{{\overset{ˆ}{\alpha }}_{L}^{w}}{{\overset{ˆ}{\alpha }}_{L0}^{w}}={\left(1+\frac{q^{\bar{\bar{w}}}}{{\overset{ˆ}{R}}^{w}}\frac{\text{d}{\overset{ˆ}{R}}^{w}}{\text{d}q^{\bar{\bar{w}}}}\right)}^{-0.697}.$$

**Figure 5.**
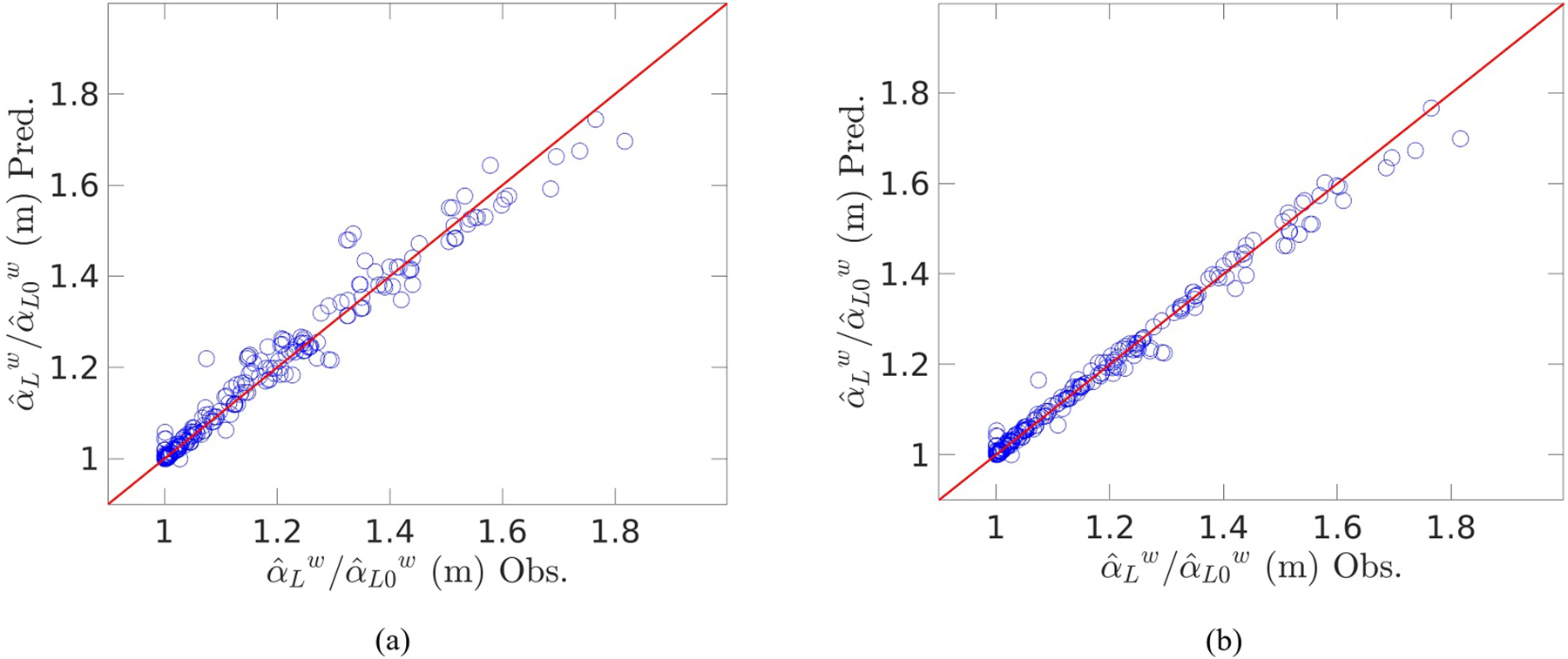
Comparison between observed and predicted dispersivity using: (a) Function 4; and (b) Function 7.

Equation 27 is relatively simple and provides an effective model for dispersivity. The average relative error observed when predicting dispersivity with this model was 1.32%, while the maximum error was 13.4%. Of the 323 simulations run here, Equation 27 predicted dispersivity with less than 1% error for 64% of the simulations, and predicted with less than 2% error for 80% of the simulations. A comparison between the predicted and observed dispersivities may be seen in Figure 5a. For completeness, the most accurate model, Function 7, was also investigated, and it was found to very accurately model the dispersivity, as can be seen in Figure 5b. Using Function 7, the average relative error observed was 0.712%, with a maximum error of 8.4%. Function 7 predicted the dispersivity with less than 2% error for 94% of the simulations.

With the dispersivity models described in Table 5, which are entirely dependent on quantities related to flow, it is possible to model DST during flow of Cross and Carreau fluids through the medium studied without running any microscale simulations for the GNF of concern so long as the Newtonian dispersivity is known. Using the equations described in Table 5 to compute ${\overset{ˆ}{\alpha }}_{L}^{w}$, the dispersion was computed from Equation 22, and then this dispersion was used to model macroscale DST using Equation 21. The RMSE observed using this predicted dispersion coefficient was only slightly higher than what was observed using the fitted dispersion coefficient, shifting by <1%.

**Figure 6.**
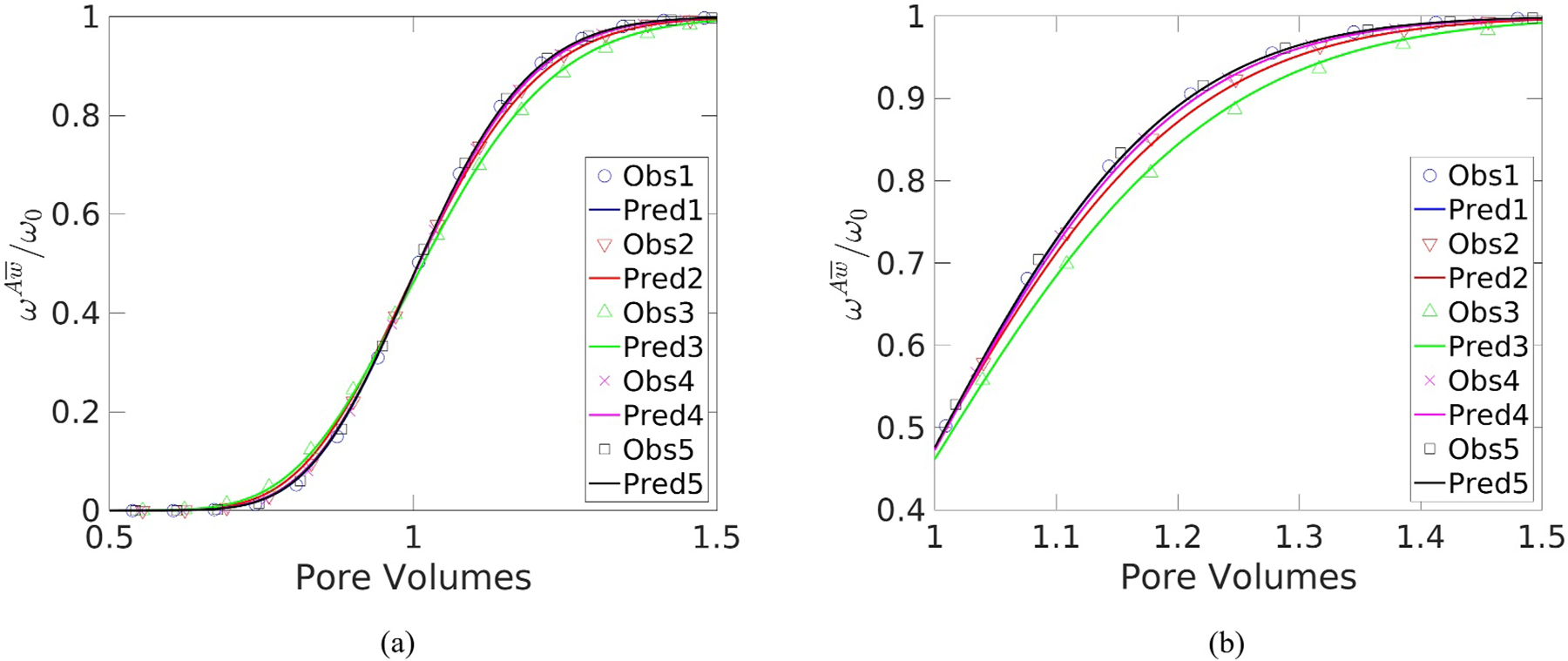
Select breakthrough curves for Carreau Fluid two in Packing: (a) at full scale; and (b) zoomed into the upper section.

Breakthrough curves generated from these macroscale model results are plotted versus the averaged microscale simulation results in Figure 6 for Carreau Fluid 2 flowing in Medium 4, which exhibited the highest error of all the fluids. The breakthrough curves were generated for the same flow rates used for Figure 3. Figure 6 shows that the macroscale model compares favorably to the microscale simulation results. The results obtained using other Functions from Table 5 did not significantly improve the RMSE compared to Function 4, indicating that this simpler function is adequate when modeling DST in this porous medium.

## 5. Discussion

Mechanistic modeling of DST in GNFs is complicated. Flow modeling has traditionally relied upon model systems such as a bundle of capillary tubes (S. Liu & Masliyah, 1999; Pearson & Tardy, 2002; Sochi, 2010; Kaur et al., 2011; Fayed et al., 2016; Castro & Radilla, 2017; Basset et al., 2019; Hauswirth et al., 2019; Castro, 2019) or empirical approaches reliant upon parameterization based upon experimental data collected with the fluid of concern (Hauswirth et al., 2020; Orgeas et al., 2006; Orgéas et al., 2007; Patacchini & de Loubens, 2013; Perrin et al., 2006; Sorbie et al., 1989; Tosco et al., 2013; Woods et al., 2003; Zhang et al., 2019). DST modeling further complicates the situation, since it is known that GNFs can exhibit more dispersion than Newtonian fluids and that result depends upon flow conditions and fluid properties (Chaplain et al., 1998; D’Onofrio et al., 2002; Saadun et al., 2021; Singh & Murthy, 2022). The goal of this work was to advance a mechanistic model for DST in GNFs flowing in unconsolidated porous medium systems. Such a solution requires a flow solution and a transport solution.

Building upon and confirming recent work (C. A. Bowers & Miller, 2021; C. A. Bowers & Miller, 2023), GNF flow was modeled accurately based upon porous medium and rheological characteristics in a predictive manner without the need for specific experiments or simulations of the GNF system of concern. This enables a prediction of resistance as a function of flow rate for any Cross or Carreau fluid, thus solving the flow portion of model.

For the transport portion of the problem, Fickian behavior was confirmed for the systems studied, which is a convenient finding and a starting point for modeling transport in GNF systems. Fickian behavior was observed for all media, fluids, and flow rates investigated, which builds upon the experimental observations of such behavior (An et al., 2022; Boschan et al., 2008). Dispersivity was observed to depend upon the macroscale velocity of the Cross and Carreau fluids in a manner that seemingly depended upon the fluid properties.

The work carried out here may be considered a comprehensive analysis of DST during flow of Cross and Carreau model fluids through the porous media studied. A model was validated for multiple porous medium systems, and it is expected that this model would apply to other systems of a similar class to those studied here. It is expected that Fickian dispersion will be observed for other porous medium systems, as has been reported in the literature (An et al., 2022; Boschan et al., 2008; Chaplain et al., 1998; D’Onofrio et al., 1997), but it is not known whether Equation 27 would apply to all classes of porous media. If the dispersivity of a medium and its scaling with GNF fluids are separable, then this would be the case. This analysis was carried out for isotropic media, and is based on classical dispersion theory that has historically been limited to isotropic systems. This being said, the flow model used to arrive at the dispersivity model presented here can and has been applied to anisotropic media, and could be used to extend this work in the future. The Buckingham Pi analysis and use of PySR to approximate the unknown function appears to be a useful path in any event—for DST in GNF systems and potentially many other applications.

A useful dispersivity model would allow one to simulate DST during GNF flow at the macroscale with a minimal number of experiments or simulations. The model developed here meets these criteria, as it only requires easily observed or approximated system and fluid properties, similar to the hydraulic resistance model developed in (C. A. Bowers & Miller, 2021; C. A. Bowers & Miller, 2023).

## 6. Summary and Conclusions

DST during GNF flow in porous media occurs in many industrial, biological, and geological applications. Despite its importance, there is a gap in the literature when it comes to modeling this transport. Past efforts have been focused on idealized fluids flowing through idealized media, such as capillary pore-networks, without consensus on modeling methods for realistic fluids flowing in media that may be found in common applications. In particular, many biological and industrial fluids may be classified as Cross or Carreau model fluids, which are more difficult to model than the power-law fluids that are typically investigated in the literature. The flow paths of species in these fluids are expected to change with the flow rate, impacting transport in ways that have not been effectively modeled before.

This work set out to determine whether Fickian dispersion applies to generalized Newtonian fluids in porous medium systems, as well as how this dispersion changes with flow rate. Eight different Cross and Carreau fluids were investigated. To model transport in these fluids, the applicability of classical Fickian dispersion theory for isotropic media was investigated. To assess whether the Fickian closure relation applied to GNF flow, microscale simulations of transport through several random packings of spheres were carried out and the averaged microscale results were used to fit a dispersion coefficient to the macroscale model. The error introduced by the Fickian assumption was quantified during this fitting process. The longitudinal dispersivity was computed from the fitted dispersion coefficients, and a machine-learning symbolic regression algorithm was used to formulate a model that could predict the dispersivity for a given flow rate.

Flows were simulated for all eight of the fluids at the microscale such that the full range of generalized Newtonian flow behavior was observed, and species transport simulations were carried out using the resulting velocity fields. It was found from the flow simulations that the hydraulic resistance models developed in (C. A. Bowers & Miller, 2021; C. A. Bowers & Miller, 2023) apply to these porous media and fluids, and the resistance model parameters were tabulated. Fickian dispersion coefficients were fit using averaged results from the microscale transport simulations and it was found that the Fickian model accurately represented the simulation results for all fluids and all velocities. The calculated dispersivities exhibited behavior that was related to the hydraulic resistance behavior of the fluids, motivating further investigation.

The dependence of dispersivity on system state properties was investigated using a Buckingham Pi analysis to non-dimensionalize potentially important variables followed by an automated symbolic regression approach, PySR, to determine a functional form predicting the dimensionless dispersivity as a function of other non-dimensional quantities. A variety of candidate functions were identified and a relatively simple function was found to provide a useful prediction of observed behavior.

The dispersivity model that resulted from this work was reliant on the hydraulic resistance model developed in (C. A. Bowers & Miller, 2021; C. A. Bowers & Miller, 2023). Coupling the hydraulic resistance model, the closed macroscale species transport model, and the dispersivity model, it is possible to accurately simulate flow and transport of other Cross or Carreau model fluids through these porous media without any additional microscale simulations.

While the set of simulations carried out here were comprehensive for this class of media, namely randomly packed non-overlapping spheres, further work is needed to determine whether the model applies to other classes of porous media, such as fractured or consolidated media. Future work will be focused on expanding this analysis to other porous media, as well as other classes of GNFs.

## Figures and Tables

**Table 1:** Sphere Pack Properties

Parameter	Packing 1	Packing 2	Packing 3	Packing 4
Number of Spheres	4,000	4,000	4,000	4,000
Domain Length in x and y(m)	$1.00\times 10^{-3}$	$1.00\times 10^{-3}$	$1.00\times 10^{-3}$	$1.00\times 10^{-3}$
Domain Length in z(m)	$8.00\times 10^{-3}$	$8.00\times 10^{-3}$	$8.00\times 10^{-3}$	$8.00\times 10^{-3}$
Porosity (${\text{m}}^{3}/{\text{m}}^{3}$)	$4.05\times 10^{-1}$	$4.05\times 10^{-1}$	$4.05\times 10^{-1}$	$4.06\times 10^{-1}$
Mean Radius (m)	$6.60\times 10^{-5}$	$6.52\times 10^{-5}$	$6.27\times 10^{-5}$	$6.00\times 10^{-5}$
Log Normal Radius Variance	0	$1.00\times 10^{-2}$	$5.00\times 10^{-2}$	$1.00\times 10^{-1}$
Number of Cells	76,234,059	75,793,417	74,010,272	71,917,433

**Table 2:** Fluid Parameters Used for Both Cross and Carreau Model Fluids

Parameter	Fluid 1	Fluid 2	Fluid 3	Fluid 4
${\overset{ˆ}{\mu }}_{0}(\text{Pa}\;\text{s})$	$1.0\times 10^{0}$	$1.0\times 10^{1}$	$1.0\times 10^{0}$	$1.0\times 10^{0}$
${\overset{ˆ}{\mu }}_{\infty }(\text{Pa}\;\text{s})$	$1.0\times 10^{-2}$	$1.0\times 10^{-2}$	$1.0\times 10^{-2}$	$1.0\times 10^{-2}$
m(s)	$1.0\times 10^{5}$	$1.0\times 10^{5}$	$1.0\times 10^{5}$	$1.0\times 10^{3}$
n	$5.0\times 10^{-1}$	$5.0\times 10^{-1}$	$7.0\times 10^{-1}$	$5.0\times 10^{-1}$
$\rho^{w}\left(\text{kg}/{\text{m}}^{3}\right)$	$1.00\times 10^{3}$	$1.00\times 10^{3}$	$1.00\times 10^{3}$	$1.00\times 10^{3}$

**Table 3:** Controls Used During PySR Analysis

Control parameter	Value
Populations	24
Population size	50
Number of iterations	$1\times 10^{4}$
Number of cycles-per-iteration	$2\times 10^{3}$
Maximum Complexity	15
Operators	+, *, /, ^
Complexity of Constants	2
Complexity of Operators	^:2
Operator Constraints	^:(−1, 2)
Nested Operator Constraints	^:{^:0}
Randomization Weight	0.01

**Table 4:** Porous Media Flow Properties

Parameter	Packing 1	Packing 2	Packing 3	Packing 4
Permeability, ${\overset{ˆ}{k}}^{w}\left({\text{m}}^{2}\right)$	$1.73\times 10^{-11}$	$1.79\times 10^{-11}$	$1.92\times 10^{-11}$	$8.49\times 10^{-12}$
Specific Interfacial Area, $\epsilon^{\bar{\bar{\text{ws}}}}\left({\text{m}}^{2}/{\text{m}}^{3}\right)$	$4.00\times 10^{4}$	$3.97\times 10^{4}$	$3.82\times 10^{4}$	$8.97\times 10^{4}$
Resistance Length Scale, ${\overset{ˆ}{L}}^{W}(\text{m})$	$1.09\times 10^{-5}$	$1.12\times 10^{-5}$	$1.16\times 10^{-5}$	$4.78\times 10^{-6}$

**Table 5:** Summary of Symbolic Regression Results From PySR.*

Function	Equation	Loss	GCV
1	y=1.04	9.41%	$4.09\times 10^{-2}$
2	$y=0.441^{x_{5}}$	1.76%	$1.78\times 10^{-3}$
3	$y=0.217^{x_{5}}+x_{5}$	1.36%	$9.53\times 10^{-4}$
4	$y={\left(1+x_{5}\right)}^{-0.697}$	1.32%	$9.37\times 10^{-4}$
5	$y={\left(\frac{-1.13}{-2.78+x_{2}}\right)}^{x_{5}}$	1.18%	$1.01\times 10^{-3}$
6	$y={\left[0.468+0.193\left(x_{2}+x_{5}\right)\right]}^{x_{5}}$	0.806%	$4.70\times 10^{-4}$
7	$y={\left[\frac{0.352}{0.752+\left(x_{2}+x_{5}\right)x_{5}}\right]}^{x_{5}}$	0.712%	$2.58\times 10^{-4}$

Note.

* Variables given to PySR were indexed such that $y={\overset{ˆ}{\alpha }}_{L}^{w}/{\overset{ˆ}{\alpha }}_{L0}^{w},x_{0}=Mq^{\bar{\bar{w}}},x_{1}={\overset{ˆ}{R}}^{w}/{\overset{ˆ}{R}}_{\infty }^{w},x_{2}={\overset{ˆ}{R}}^{w}/{\overset{ˆ}{R}}_{0}^{w},x_{3}=n_{1},x_{4}=n_{2}$, and $x_{5}=\left(q^{\bar{\bar{w}}}/{\overset{ˆ}{R}}^{w}\right)\text{d}{\overset{ˆ}{R}}^{w}/\text{d}q^{\bar{\bar{w}}}$.

## Data Availability

OpenFOAM is an open-source software that is readily available (Greenshields, 2018). PySR is available for free from the GitHub referenced in (Cranmer, 2023). Data used to generate the figures for this work are available at (C. Bowers & Miller, 2024).

## Key Points:

- Macroscale flow of Cross and Carreau model fluids was predicted based upon theoretical results and shown to agree with simulation data
- In the Fickian limit, dispersion is shown to depend upon the flow velocity for generalized Newtonian fluids
- A macroscale dispersivity model was derived using symbolic regression that could be used to accurately model species transport
